# Understanding the Shade Tolerance Responses Through Hints From Phytochrome A-Mediated Negative Feedback Regulation in Shade Avoiding Plants

**DOI:** 10.3389/fpls.2021.813092

**Published:** 2021-12-22

**Authors:** Huiying Xu, Peirui Chen, Yi Tao

**Affiliations:** Key Laboratory of Xiamen Plant Genetics and State Key Laboratory of Cellular Stress Biology, School of Life Sciences, Xiamen University, Xiamen, China

**Keywords:** shade tolerance, shade avoidance, phytochrome A, phytohormones, *Arabidopsis*

## Abstract

Based on how plants respond to shade, we typically classify them into two groups: shade avoiding and shade tolerance plants. Under vegetative shade, the shade avoiding species induce a series of shade avoidance responses (SARs) to outgrow their competitors, while the shade tolerance species induce shade tolerance responses (STRs) to increase their survival rates under dense canopy. The molecular mechanism underlying the SARs has been extensively studied using the shade avoiding model plant *Arabidopsis thaliana*, while little is known about STRs. In *Aarabidopsis*, there is a PHYA-mediated negative feedback regulation that suppresses exaggerated SARs. Recent studies revealed that in shade tolerance *Cardamine hirsuta* plants, a hyperactive PHYA was responsible for suppressing shade-induced elongation growth. We propose that similar signaling components may be used by shade avoiding and shade tolerance plants, and different phenotypic outputs may result from differential regulation or altered dynamic properties of these signaling components. In this review, we summarized the role of PHYA and its downstream components in shade responses, which may provide insights into understanding how both types of plants respond to shade.

## Introduction

Plants grown in complex and dynamic light environments are constantly in fierce competition with their surrounding neighbors for limited light sources. Once light reaches plant leaves, it is absorbed by chlorophylls and other pigments of the photosynthetic apparatus and used for photosynthesis. The upper leaves preferentially absorb blue (B, λ = 400–500 nm) and red (R, λ = 600–700 nm) light for photosynthesis while reflecting most of the far-red (FR) (λ = 700–800 nm) light (Vandenbussche et al., 2005; Franklin, 2008). As a result, the shading of upper leaves not only reduced the total intensity but also the R:FR ratio of light reaching lower leaves (Franklin, 2008). The reduction in R:FR serves as an indicator of local vicinity of vegetation (Vandenbussche et al., 2005; Franklin, 2008) and is perceived by phytochromes, a family of R\FR photoreceptors.

Depending on the strategy that is adopted by plant to cope with vegetative shade, we typically classify plants into two groups: shade avoiding or shade tolerance species. In response to shade, plants living in open habitats often induce a series of shade avoidance responses (SARs) to outgrow their competitors. On the other hand, species typically found in forest understories exhibit shade tolerance responses (STRs), which allows them being adapted to shade environment. The molecular mechanism that underlies the SARs have been extensively studied, while little is known about the STRs. Recent studies indicated that during the SARs, several negative regulators of the SARs, including PHYTOCHROME A (PHYA), were induced. PHYA functions to tune down the SARs under excessive or prolonged shade conditions, which may allow the shade avoiding species to become tolerant to shade (Song et al., 2020). Interestingly, PHYA was also reported to suppress hypocotyl elongation in *Cardamine hirsuta*, a shade tolerance plant (Molina-Contreras et al., 2019). It is thus possible that some signaling components are shared between the shade avoiding and shade tolerance species. In this review, we summarize results from recent studies on the role of PHYA in regulating low R:FR induced SARs, which may provide hints for understanding the potential mechanism of the STRs.

## The Shade Avoidance Responses

The SARs encompass various phenotypic traits, including elongation of stems and petioles, upward leaf movement (increased hyponasty), enhanced apical dominance and reduced branching, which allows plants to reach out for more light (Morgan et al., 1980; Johnson, 1982; Franklin and Whitelam, 2005; Mullen et al., 2006; Franklin, 2008). These responses come at the cost of reduced development of leaf, root and storage organs, such as reduced leaf area, chlorophyll content and chlorophyll a:b ratio (Smith and Whitelam, 1997). Under prolonged shade, flowering is accelerated to allow seed set (Halliday et al., 1994; Casal, 2012). Furthermore, SARs are also accompanied by reduced resistance to various pathogens and symbiotic interactions with microorganisms (Moreno et al., 2009; Cerrudo et al., 2012; De Wit et al., 2013; Ballare, 2014; Konvalinková and Jansa, 2016).

The R/FR light signal is perceived by the phytochrome family of photoreceptors. Three major types of phytochromes, PHYA, PHYB, and PHYC, were identified in angiosperms (Sharrock and Quail, 1989). In *Arabidopsis thaliana*, there are five phytochromes (PHYA-PHYE) (Sharrock and Quail, 1989; Clack et al., 1994). Among them, PHYB is the major phytochrome repressing SARs under sun light. *phyB* mutants display constitutive shade avoidance phenotype (Somers et al., 1991; Devlin et al., 1992; Reed et al., 1993; Robson et al., 1993; Kerckhoffs et al., 2010). *phyA* mutant, on the other hand, displayed exaggerated hypocotyl extension in response to low R:FR signal, but was indistinguishable from the wild type under continuous white light (Johnson et al., 1994). PHYA is thus believed to mediate the negative feedback regulation of the SARs.

## The Shade Tolerance Responses

In contrast to the well-defined SARs, the STRs vary a lot between species and are influenced by plant ontogeny and various biotic and abiotic factors (Valladares and Niinemets, 2008). The shade tolerance species usually have reduced elongation of stem and petiole in shade, as compared to the shade avoidance species (Smith, 1979; Niinemets, 1997; Niinemets and Valladares, 2004). However, shade tolerance is not just a lack of shade avoidance. Shade tolerance species induce STRs to optimize their survival under shade, such as lower growth rates, having thinner leaves, altered chlorophyll a:b ratio, reduced apical dominance (increased branching) (Beneragama and Goto, 2011; Casal, 2012; Gommers et al., 2013; Roig-Villanova and Martinez-Garcia, 2016). Several hypotheses have been proposed to explain the survival strategy of the shade tolerance species. The “carbon gain hypothesis” proposed that the shade tolerance may be achieved through optimizing light capture and lowering dark respiration rate, and therefore increasing maximum potential carbon gain (Givnish, 1988; Valladares and Niinemets, 2008). For example, shade tolerant species can optimize light capture and utilization by increasing PSII (photosystem II):PSI ratio (Melis and Harvey, 1981) and special leaf area [SLA, leaf area (m^2^) per leaf dry mass (kg)], lowering chlorophyll a:b ratio (Melis and Harvey, 1981; Evans and Poorter, 2001). Other studies suggested that it is the pattern of the relationship between leaf nitrogen and irradiance (Niinemets, 1997) or the relative growth rate (Reich et al., 1998) that correlates with the shade tolerance. An extension of the carbon gain hypothesis is the “trade-off hypothesis,” which predicts an inverse correlation between growth rates in high light and survival rates in low light (Valladares and Niinemets, 2008). The “Stress tolerance hypothesis,” on the other hand, proposed that survival in shade positively correlates with resistance to biotic and abiotic stress (Kitajima, 1994). Shade tolerance correlates positively with resistance to pathogens and diseases and increasing physical defense to the external environment (Augspurger, 1984). In deciduous trees, it was also found that the shade-tolerant species restrict carbon allocation toward defense and radial growth instead of increasing height and storage capacities (Giertych et al., 2015). In shade avoiding *Arabidopsis*, mutant that failed to induce elongation growth in shade still exhibited FR-induced attenuation in defense response, suggesting alteration in defense is not a simple trade-off response (Ballare, 2014; Moreno and Ballare, 2014). In summary, the STRs may be much more complex than the SARs, as different strategies/responses may be employed by plant species with variable shade tolerance ability, or by plants at different developmental stages, or by plants facing shade along with other stresses (Valladares and Niinemets, 2008). So far, there is limited number of studies on the molecular mechanisms of the STRs.

## Phytochrome A-Mediated Inhibition of Shade Avoidance Responses

In nature, there are different degrees of shade. Light with reduced R:FR ratio can be generated under three situations: light reflection from nearby plants [neighbor detection, no direct vegetative shade, and little reduction in photosynthetically active radiations (PARs)]; direct, but unclosed shade (mild shade, modest reduction in R:FR and PARs); and dense canopy shade (strong shade, low R:FR, and PARs) (Casal, 2012; Martinez-Garcia et al., 2014). Most shade-tolerant plants are herbaceous species that live in the understory of the forest and usually receive strong canopy shade. For shade avoiding plants grown under dense canopy, the elongation growth of stems, a typical SAR, is also inhibited in a PHYA-dependent manner (Martinez-Garcia et al., 2014; Yang et al., 2018; Song et al., 2020). By measuring the hypocotyl growth rates under mild (R:FR ca. 0.7) and strong shade (R:FR ca. 0.1), Song et al. (2020) demonstrated that PHYA mainly functions after prolonged shade (24 h) and under very low R:FR ratio light. Through whole genome expression profiling, Devlin et al. (2003) also showed that there was an antagonistic effect of PHYA after prolonged shade treatment (24 h). Furthermore, PHYA is also involved in repressing FR-dependent senescence and leaf yellowing in shade (Brouwer et al., 2014; Lim et al., 2018). *phyA* mutant had reduced survival in deep vegetative shade (Yanovsky et al., 1995). Both the transcript and protein level of PHYA increased after low R:FR treatment, nuclear localization of PHYA was also enhanced by prolonged strong shade treatment (Devlin et al., 2003; Yang et al., 2018; Song et al., 2020). Thus, in shade avoiding plants, PHYA is activated by shade to inhibit some of the SARs and to prevent exaggerated responses to shade, especially under dense canopy.

Theoretically, it is plausible for shade-tolerant plants to develop some of the STRs through enhancing PHYA signaling pathway. Over-expressing PHYA in tomato, tobacco, rice, potato and creeping bentgrass, and zoysiagrass all inhibited stem elongation in shade (McCormac et al., 1992; Robson et al., 1996; Rousseaux et al., 1997; Kong et al., 2004; Garg et al., 2006; Ganesan et al., 2012). Recent studies showed that PHYA protein in a shade-tolerant plant *C. hirsuta* (a close relative of *Arabidopsis*) has stronger activity in inhibition of hypocotyl elongation than that of the *Arabidopsis* (Jose Molina-Contreras et al., 2019). It was reported that a single amino-acid change in PHYA caused a 100-fold shift in the threshold for FR light sensitivity (Maloof et al., 2001). Thus, natural variations in PHYA may allow plants to activate PHYA-mediated negative feedback regulation with different dynamics and to cope with a wide range of shade conditions. By comparing PHYA sequence in species with different degrees of shade tolerance may help us to understand if this a widely adopted strategy in regulating shade responses.

## Constitutive Photomorphogenic 1 is a Key Regulator of the Phytochrome A Pathway

Under shade, PHYA was proposed to act through the FR-high irradiance response (Martinez-Garcia et al., 2014) and it may attenuate the SARs through antagonizing PHYB signaling (Johnson et al., 1994; Ciolfi et al., 2013). Results from the genome wide expression profiling suggested that PHYA acts partially through antagonizing the effect of PHYB, while there also exist shade responsive genes that are specifically targeted by PHYA alone (Devlin et al., 2003). Song et al. (2020) reported that shade-induced nuclear localization of COP1 (CONSTITUTIVE PHOTOMORPHOGENIC 1), a key negative regulator of photomorphogenesis, was suppressed in a PHYA-dependent manner after prolonger shade. Mutation in *COP1* fully suppressed the long hypocotyl phenotype of *phyA* under strong shade, indicating it is a key regulator in PHYA-mediated elongation growth. COP1 interacts with the suppressor of *phyA-105* (SPA) proteins and functions as an E3 ubiquitin ligase that promotes the ubiquitination and degradation of target proteins, including negative regulators of the SARs such as HY5 (ELONGATED HYPOCOTYL 5), HFR1 (LONG HYPOCOTYL IN FAR-RED) and PAR1,2 (PHYTOCHROME RAPIDLY REGULATED 1,2) and BBX21,22 (B-box-containing proteins 21,22) (Ang and Deng, 1994; Holm et al., 2002; Saijo et al., 2003; Jang et al., 2005; Zhu et al., 2008; Crocco et al., 2010; Zhou et al., 2014). Indeed, comparing HY5 protein level in the strong shade vs. that in the mild shade, a PHYA-dependent increase was observed. As COP1 acts downstream of both PHYA and PHYB, it may account for the antagonizing effect of PHYA.

## Regulation of Phytochrome Interacting Factors by Phytochrome A

PHYTOCHROME INTERACTING FACTOR (PIF) transcriptional factors play a key role in regulating the expression of shade responsive genes and elongation growth. Mutation in *PIF4,5,7* led to reduced SARs, including shade-induced hypocotyl elongation and response of shade marker genes. PIF4,5,7 accumulated rapidly after low R:FR treatment, which is proceeded by changes at protein phosphorylation level (Lorrain et al., 2008; Hornitschek et al., 2009, 2012; Li et al., 2012; Huang et al., 2018). After prolonged strong shade treatment, PIF4 protein level decreased in a PHYA-dependent manner (Song et al., 2020). Mutation in *PIF4,5* partially suppressed the exaggerated hypocotyl growth of *phyA* mutant in strong shade, and the altered expression of PHYA downstream target genes (Song et al., 2020).

**FIGURE 1:**
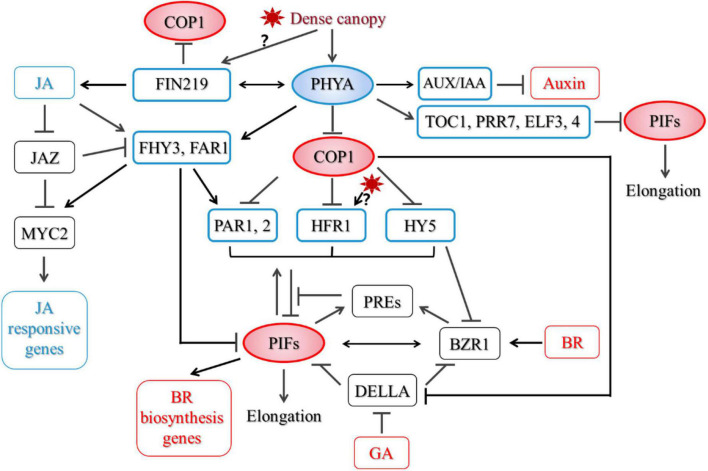
A proposed model for phytochrome A (PHYA)-mediated regulatory network in shade. Proteins outlined in blue are negative regulators of the shade avoidance responses (SARs), while those in red are positive regulators.

Little is known about how PHYA regulates PIF after prolonged strong shade treatment. Based on previous studies, we speculate that under strong shade, PHYA may regulate the stability, modification and activity of PIFs. First, COP1 is required for accumulation of PIF3 in darkness (Bauer et al., 2004). Ling et al. (2017) reported that COP1/SPA complex associates with and stabilizes PIF3 through repressing BIN2 (BRASSINOSTEROID-INSENSITIVE 2)-mediated phosphorylation of PIF3 and its subsequent degradation. Thus, Shade-activated PHYA may regulate the protein level of PIFs through COP1-dependent route. But not all PIFs are degraded under FR light. PIF8 is degraded in a COP1-dependent manner in dark. Under FR light, activation of PHYA led to stabilization of PIF8, which inhibits suppression of hypocotyl growth by PHYA in FR light (Oh et al., 2020). Whether PIF8 is involved in PHYA-mediated SARs remains to be investigated. Secondly, PHYA preferentially interacts with PIF1 and PIF3 in its Pfr form. In most cases, the interaction led to phosphorylation of PIFs and its subsequent degradation (Shen et al., 2005, 2007, 2008; Al-Sady et al., 2006). An *Avena sativa* phytochrome A (AsPHYA) was shown to directly phosphorylate PIF3, and a mutant version of AsPHYA with reduced kinase activity compromised FR-induced phosphorylation and degradation of PIF3 (Shin et al., 2016). Finally, shade induces the expression of several PIF-interacting proteins, such as HFR1, PAR1,2, which represses the transcriptional activity of PIFs (Roig-Villanova et al., 2007; Hornitschek et al., 2009; Galstyan et al., 2011; Hao et al., 2012). PHYA does not affect early induction of *PAR1* and *HFR1* by shade. After prolonged shade treatment, PHYA partially suppresses *PAR1* expression, but does not affect *HFR1* expression (Song et al., 2020). Thus, PHYA does not inhibit SARs through upregulating the transcript level of *PAR1* and *HFR1.* FHY3 (FAR-RED ELONGATED HYPOCOTYL3) and FAR1 (FAR-RED-IMPAIRED RESPONSE1) are two homologous transcription factors that act downstream of PHYA in FR light signaling (Wang and Deng, 2002; Lin et al., 2007). *fhy3far1* mutant displayed exaggerated hypocotyl elongation under shade, which was similar to *phyA* (Liu et al., 2019). They also directly interact with PIF3,5 and repress their transcriptional activity (Liu et al., 2020). It would be interesting to see if FHY3 and FAR1 mediate PHYA signaling in shade and how they are regulated. Recently, Fraser et al. (2021) showed that early evening expression of the central circadian clock components *TOC1* (TIMING OF CAB EXPRESSION 1), *PRR7* (PSEUDO RESPONSE REGULATOR 7), and *ELF3,4* (EARLY FLOWERING 3,4) was elevated in photocycles of low R:FR and low PAR in a PHYA-dependent manner. And among them, TOC1, PRRs, and ELF3 also interact with PIFs and negatively regulate their transcriptional activities and/or DNA binding ability (Nieto et al., 2015; Soy et al., 2016; Martin et al., 2018; Jiang et al., 2019; Zhang et al., 2020). Together, these proteins suppress stem elongation under strong shade (Figure 1).

Thus, in addition to affecting PIFs through up-regulating PHYA level and/or activity, shade tolerance plants may also tune down the activity of PIFs through a PHYA-independent pathway, either through having PIFs with altered protein dynamics or activity, or by using negative regulators that can strongly reduce the activity or protein level of PIFs. Ciolfi et al. (2013) demonstrated that although in *Arabidopsis*, both PHYA and HFR1 are negative regulators of SARs, they act independently in regulating several shade-induced target genes. HFR1 was also shown to regulate shade-induced flowering independent of PHYA. HFR1 interacts with CO (CONSTANS) and PIF7, which inhibit their DNA binding activities to downstream target genes (Zhang et al., 2019). It was reported that HFR1 in the shade tolerant *C. hirsuta* was more stable than its counterpart in *Arabidopsis* due to its lower binding capacity to COP1. The enhanced HFR1 activity then caused a reduction in PIFs’ activity and attenuated PIF-mediated responses (Paulisic et al., 2021). Furthermore, it was reported that the shade avoiding Scots pine lacks FR high irradiance response, suggesting a different negative regulatory pathway in angiosperm, if there is any (Mohr, 1990).

## Phytohormones and the Phytochrome A Signaling Pathways

In shade avoiding plants, changes in plant architecture, defense responses and metabolism are associated with both altered level and sensitivity of various phytohormones as previously reviewed (Yang and Li, 2017; Fernandez-Milmanda and Ballare, 2021; Liu et al., 2021). For example, in *Arabidopsis*, shade induced stem elongation requires growth promoting hormones: auxin, gibberellins (GAs) and brassinosteroids (BRs), while shade reduced expression of defense genes that are responsive to jasmonic acid (JA) and salicylic acid (SA). Here we summarize phytohormones that are influenced by PHYA under shade.

Biosynthesis of auxin increases in shade. Mutation in *TAA1* (Trp aminotransferase in Arabidopsis), a key enzyme in Trp-dependent auxin biosynthesis, severely impaired hypocotyl elongation in shade (Tao et al., 2008). However, this mutation had limited effect on hypocotyl growth in *phyA* mutant, suggesting a TAA1-independent mechanism may be employed by PHYA. Both PHYA and PHYB can interact with a similar set of AUX/IAA (auxin/indole-3-acetic acid) proteins, and PHYA activated under deep shade can thus counteract the effect of PHYB through stabilizing these proteins and subsequently suppressing the auxin sensitivity and the SARs (Xu et al., 2018; Yang et al., 2018).

Both BR biosynthesis and signaling components are required for petiole and stem growth under low R/FR (Kim et al., 1998; Luccioni et al., 2002; Kozuka et al., 2010; Keller et al., 2011). Activation of the BR signal pathway lead to the dephosphorylation of BES1/BZR1 transcriptional factors, which then move into the nucleus and promote the expression of BR-responsive genes (Belkhadir and Jaillais, 2015). PHYA inhibits hypocotyl elongation partially through repressing key BR biosynthesis genes’ expression in hypocotyls, which is mediated by COP1, PIF4, and PIF5 (Song et al., 2020). In addition, BES1/BZR1 directly interacts with PIF4, they regulate downstream target genes and promote hypocotyl elongation interdependently in response to BR, darkness and heat (Oh et al., 2012). HY5 also directly interacts with the active form of BZR1 and attenuates its transcriptional activity (Li and He, 2016). HY5 represses BZR1 accumulation through enhancing BIN2 kinase activity (Li et al., 2020). After prolonged strong shade treatment, protein level of both PIF4 and the active form of BES1 were much higher in *phyA* mutant than those in the wild type, while HY5 protein level was repressed by PHYA (Song et al., 2020). Thus, PHYA may also repress BR signaling through regulating BES1/BZR1-interacting proteins (Figure 1).

Although little is known about how PHYA affects GA pathway, the dwarf phenotype of PHYA overexpressing-tobacco was related to a reduction in the active GA level (Jordan et al., 1995). DELLA proteins are transcriptional regulators that inhibit GA signaling. DELLAs are also targets of COP1, which promotes degradation of DELLAs through direct protein interactions and ubiquitination (Blanco-Tourinan et al., 2020). Furthermore, DELLAs interact with BZR1 and PIFs to repress their activities (Feng et al., 2008; Bai et al., 2012). BZR1 and PIF4 share common target genes, including PRE1 (PACLOBUTRAZOL RESISTANCE1), which is co-regulated by BR, GA, and auxin (Bai et al., 2012). By forming heterodimers with PAR1, PRE1 prevents PAR1 from interacting with PIF4 and represses its activity (Hao et al., 2012). These findings defined a regulating network including DELLAs, BZR1, PIFs, and downstream targets, which can integrate GA and BR signaling with light signal to fine-tune SARs. How this signaling network is influenced by PHYA and in the shade tolerance species remain to be illustrated.

Jasmonic acid is a lipid-derived plant hormone that is well known for its involvement in responses to wounding, necrotrophic pathogens and herbivores. Furthermore, JA participates in plant development regulation, including root growth inhibition, trichome initiation, male fertility, leaf senescence and photomorphogenesis. JA suppresses hypocotyl elongation and promotes cotyledon opening through inhibiting COP1 activity (Zheng et al., 2017). In *Arabidopsis*, both JA biosynthesis and sensitivity is reduced in shade due to inactivation of PHYB (De Wit et al., 2013; Leone et al., 2014; Fernandez-Milmanda et al., 2020). *phyA* seedlings grown in dark and FR contains higher level of OPDA [*cis*-(+)-12-oxophytodienoic acid], an intermediate of JA biosynthesis, than wild type (Robson et al., 2010). In addition, PHYA is required for inhibition of root growth by JA, and wound/JA-induced degradation of JAZ1 (JASMONATE-ZIM-DOMAIN PROTEIN 1), repressors of JA signaling (Robson et al., 2010), indicating that PHYA is also required for JA signaling. *Arabidopsis* FIN219 (FAR-RED INSENSITIVE219)/JAR1 (JASMONATE RESISTANT 1) is involved in PHYA-mediated FR light signaling (Hsieh et al., 2000). It encodes an enzyme catalyzing the final step of JA-Ile (an active form of JA) production. *Jar1* mutant was hypersensitive to shade-induced hypocotyl elongation (Robson et al., 2010). In seedlings grown in FRc (continuous FR), FIN219/JAR1 directly interacts with COP1 and retains COP1 in the cytoplasm, which subsequently increases HY5 protein level (Wang et al., 2011). Under shade, FIN219/JAR1 protein level is reduced. It affects shade-induced accumulation of PHYA protein and shade-regulated expression of *PIF5*, *PAR1* and the auxin responses gene *IAA29* and *SAUR68* (Swain et al., 2017). Interestingly, through analyzing *phyAfin219* double mutants, it was proposed that FIN219 and PHYA synergistically regulating shade-induced hypocotyl elongation and gene expression, and FIN219-mediated repression of SARs is independent of PHYA-mediated high irradiance response (Swain et al., 2017). Thus, FIN219 may serve as a key node that can sense PHYA-independent shade signal and regulates downstream components of the PHYA signaling pathway.

Phytochrome A signaling components FHY3 and FAR1 were shown to repress hypocotyl growth through direct transcriptional activation of PAR1 and PAR2 and their transcriptional activities were attenuated by JAZ proteins through direct protein interactions (Liu et al., 2019). Interestingly, JA also promoted the accumulation of FHY3 protein and unleashed them from inhibition by JAZ proteins (Liu et al., 2019). FHY3 interacts with MYC2, a key transcription factor in the JA pathway, enhances its transcriptional activity on the expression of JA-responsive genes, and affects defense responses (Liu et al., 2019). FHY3 and FAR1 were thus proposed to be regulators that balance growth and defense. As shade rapidly induced accumulation of FHY3 protein (Liu et al., 2019), but repressed JA biosynthesis, a detailed characterization on the dynamics of FHY3 protein level in shade and how PHYA is involved is required to fully illustrate the role of FHY3. In addition to regulating hypocotyl growth, FHY3 and FAR1 are involved in an array of SAR-related biological processes, including senescence, starch metabolism, chloroplast development, circadian gating, shoot branching, flowering control etc. (McCormac et al., 1992; Allen et al., 2006; Stirnberg et al., 2012; Tang et al., 2012; Ma et al., 2016, 2017; Liu et al., 2019, 2020; Tian et al., 2020; Xie et al., 2020a,b). It would be interesting to examine if these processes are also inhibited under prolonged strong shade and how PHYA and FHY3, FAR1 are involved (Figure 1).

Through studying a shade-avoiding and a shade tolerance species of genus *Geranium*, Gommers et al. (2018) found that auxin and GA levels, but not BR, increased in elongating petioles through local perception of FR light in the shade-avoiding species, which did not occur in the shade-tolerance species (McCormac et al., 1992). In the shade-avoiding species, BR level decreased in petioles after long exposure (11.5 h) to FR-enriched light, it would be interesting to know if this reduction in BR and the lack of induction in auxin and GA level is PHYA-regulated. Transcriptional profiling data revealed up-regulation of ethylene and cytokinin pathway genes in shade tolerance Swarnaprabha rice (Panigrahy et al., 2019). In *Arabidopsis*, shade arrests leaf primordia development is associated with an auxin-dependent induction of *AtCKX6*, a gene encoding a cytokinin oxidase involved in cytokinin breakdown (Carabelli et al., 2007). Cytokinin level is reduced after shade treatment in soybean (Wu et al., 2017). The higher rate of panicle emergence of Swarnaprabha rice in shade may thus be related to its upregulated cytokinin pathway. Shade treatment increases ethylene level and promotes petiole elongation in *Arabidopsis* (Pierik et al., 2009). Similar to JA, ethylene also plays an important role in various defense and stress responses. How ethylene affects the STRs and survival of the shade tolerance species remains to be investigated.

## Conclusion Remarks

Most of the crops are shade avoiding plants. When planted at high density, SARs are induced, which is often detrimental to yield (Smith, 1995; Duvick, 1997; Kebrom and Brutnell, 2007). Shade tolerance plants are naturally evolved to be adapted to grow under dense canopy. STRs are induced to achieve efficient light utilization and high survival rate under prolonged strong shade. Thus, suppressing SARs alone may not be sufficient to enhance the performance of crops planted at high density. Recent studies indicate that the shade avoiding plants processes a PHYA-mediated negative feedback regulation, which suppresses some of the SARs and may increase the survival of these plants under prolonged strong shade. Furthermore, some shade tolerance plants may also utilize the PHYA-mediated pathway to regulate their responses to shade. Understanding how the PHYA signaling pathway is regulated in both types of plants, what are the downstream components, and besides elongation growth, what and how other physiological processes are being regulated, can help us understand how plants survive under dense canopy. Modifying the shade responses of various crops using their existing components may be a shortcut to obtain high-yield shade tolerance crops.

## Author Contributions

HX and YT designed the opinion. HX, PC, and YT jointly wrote the opinion. All authors contributed to the article and approved the submitted version.

## Conflict of Interest

The authors declare that the research was conducted in the absence of any commercial or financial relationships that could be construed as a potential conflict of interest.

## Publisher’s Note

All claims expressed in this article are solely those of the authors and do not necessarily represent those of their affiliated organizations, or those of the publisher, the editors and the reviewers. Any product that may be evaluated in this article, or claim that may be made by its manufacturer, is not guaranteed or endorsed by the publisher.
